# Skin Structure, Physiology, and Pathology in Topical and Transdermal Drug Delivery

**DOI:** 10.3390/pharmaceutics16111403

**Published:** 2024-10-31

**Authors:** Sofia Brito, Moonki Baek, Bum-Ho Bin

**Affiliations:** 1 School of Advanced Materials Science and Engineering, Sungkyunkwan University, Suwon 16419, Republic of Korea; sofia@skku.edu; 2 Research Center for Advanced Materials Technology, Sungkyunkwan University, Suwon 16419, Republic of Korea; 3 Department of Applied Biotechnology, Ajou University, Suwon 16499, Republic of Korea; mkbaek@ajou.ac.kr; 4 Department of Biological Sciences, Ajou University, Suwon 16499, Republic of Korea

**Keywords:** skin structure, skin physiology, skin disease, skin aging, skin models, transdermal drug delivery

## Abstract

Several industries are increasingly focused on enhancing the delivery of active ingredients through the skin to optimize therapeutic outcomes. By facilitating the penetration of active ingredients through the skin barrier, these enhancers can significantly improve the efficacy of various formulations, ranging from skincare products to therapeutic agents targeting systemic circulation. As the understanding of skin physiology and the mechanisms of drug absorption deepen, these industries are adopting permeation enhancers more widely, ultimately leading to better patient outcomes and expanded treatment options. However, the structure and physiological function of the skin can vary according to different factors, such as the area of the body and between individuals. These variations, along with external environmental exposures, aging and pathological conditions, introduce complexities that must be carefully considered when designing effective delivery systems. Considering the intricacies of skin structure and physiology, tailoring systems to account for regional differences, individual variability, and changes induced by environmental factors or disease is critical to optimizing therapeutic outcomes. This review discusses the features of skin structure, physiology, and pathologies, as well as the application of permeation enhancers in these contexts. Furthermore, it addresses the use of animal skin models in transdermal delivery and dermatological studies, along with the latest developments in this field.

## 1. Introduction

The pursuit of enhanced drug delivery through the skin has gained considerable prominence across a range of industries, particularly in the fields of cosmeceuticals and medical applications [1]. As these sectors increasingly recognize the importance of optimizing therapeutic outcomes, the role of permeation enhancers has become of paramount importance. These enhancers facilitate the penetration of active ingredients through the skin barrier, which is primarily composed of the stratum corneum, thereby improving the efficacy of a wide range of formulations. In the cosmeceutical and dermatological industries, for instance, the incorporation of permeation enhancers can significantly amplify the effectiveness of skincare products, allowing for deeper and more effective delivery of bioactive compounds that address skin concerns such as aging and hydration [2,3]. Similarly, in the medical, the application of advanced drug delivery systems utilizing permeation enhancers can enhance the therapeutic effects of medications, particularly in transdermal patches and topical formulations aimed at systemic circulation [4]. By employing innovative technologies and formulations, these industries are capable of transforming the field of drug delivery, ensuring that patients and consumers alike benefit from more effective therapeutic interventions.

While topical drug delivery refers to the treatment of a localized area of the skin, transdermal drug delivery is a method designed to deliver drugs through the skin and into the systemic circulation for therapeutic effects throughout the body [5,6,7,8]. Transdermal drug delivery systems have gained recognition by offering a number of advantages, which contribute to their status as a highly attractive form of treatment. For example, this treatment approach is capable of circumventing the degradation that occurs in the gastrointestinal tract. This avoids the degradation of the therapeutic agent that would otherwise result from first-pass metabolism and the interference of pH, enzymes, and bacteria from the intestine. Furthermore, the release of the drug can be regulated according to the patient’s needs, allowing for a controlled release over a longer period of time. It is also noteworthy that this approach results in enhanced patient compliance, with minimal discomfort or inconvenience, which can be of particular importance in the context of juvenile or elderly patients.

However, both topical and transdermal drug deliveries are hindered by certain disadvantages, such as slow treatment speed and the potential for skin irritation, depending on the skin’s condition. The most significant limitation is the inherent skin barrier, which serves to prevent the penetration of potentially harmful substances into the body [9,10]. Therefore, the advancement of drug delivery technologies through the skin relies not only on the development of new pharmaceuticals but also on the enhancement of techniques that enable the successful permeation of drugs across the skin barrier. In addition, the skin may exhibit variations according to specific regions and each individual. For instance, different parts of the body show different characteristics that may require specific considerations when designing novel strategies. In addition, the skin’s constant exposure to environmental factors can lead to various insults that alter its properties and compromise its barrier function. These external factors, combined with inherent conditions like aging or skin diseases, can cause significant changes in the skin’s structure that may also impact the effectiveness of transdermal drug delivery systems. Given these complexities, it is crucial to carefully consider these factors during the design and development of drug delivery systems. Tailoring these systems to account for variations in skin characteristics across different body sites, as well as accommodating changes due to environment or insult, is essential for optimizing drug delivery and achieving therapeutic efficacy.

## 2. Fundamental Notions

### 2.1. Basic Skin Structure

**Figure 1:**
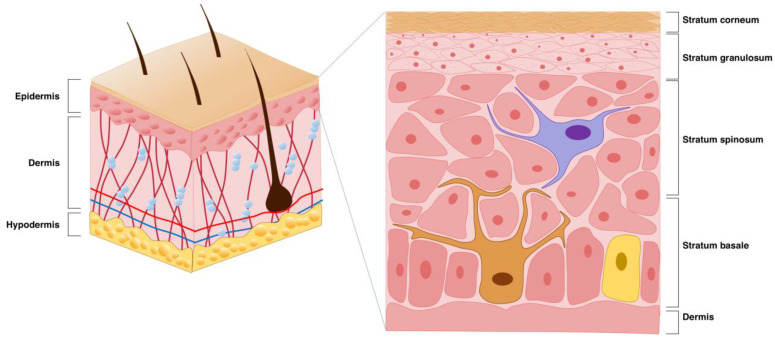
Scheme depicting the basic skin structure with particular focus on the epidermis (Not labeled in figure: purple cell (top) = Langerhans cell; brown cell (bottom left) = Melanocyte; yellow cell (bottom right) = Merkel cell).

Starting from the outermost layer, the skin is essentially composed of the epidermis, dermis, and hypodermis (Figure 1) [11,12,13,14]. The human epidermis typically consists of about 40 to 50 layers of stacked squamous epithelial cells, primarily derived from keratinocytes, which is the main cell type found in the epidermis. The epidermis is generally formed by four layers: the stratum basale, stratum spinosum, stratum granulosum, and stratum corneum. The deepest layer of the stratum basale is composed of progenitor cells, which are the youngest and most undifferentiated keratinocytes, which continuously renew the epidermis. During differentiation, human keratinocytes may take 30–40 days to migrate from the basal layer to the skin surface to undergo desquamation [15]. Merkel cells can also be found in the basal layer, as well as melanocytes, the cells in charge of pigment production [16,17]. When keratinocytes divide vertically based on the basement membrane, they contribute to keratin formation through differentiation, and when they divide horizontally, they participate in wound recovery through proliferation. This layer is separated from the dermis through a basement membrane named basal lamina. The stratum spinosum and stratum granulosum are composed of nucleated keratinocytes. These two strata comprehend about 15 to 20 layers, and the stratum granulosum has many granules accounting for high levels of keratin synthesis. Langerhans cells can be found in all layers of the epidermis and are more frequent in the stratum spinosum. As the nucleus and intracellular organelles gradually decompose, keratin accumulates, forming the stratum corneum. As keratinization progresses, keratin is further accumulated, generating a flexible and strong skin barrier composed of dead keratinocytes. The stratum corneum typically forms a 10–20 μm thick layer of keratinized keratinocytes, posing a great challenge for the topical delivery of molecules [18]. The dermal layer, located below the epidermis, is mostly composed of connective tissue constituted of fibers such as collagen and elastin [19]. The fibroblasts that produce these fibers are sparse, contrarily to the elevated numbers of keratinocytes required in the epidermis. Below the dermal layer, the hypodermis is an area composed of adipose tissues. Similarly to the dermis, the tissues contained in this layer are derived from the mesoderm. Therefore, mesenchymal stem cells present in the fat layer can differentiate into fibroblasts. Blood vessels are mainly distributed in the dermis and hypodermis but, in some cases, they extend into the epidermis. For instance, in areas containing skin growth such as warts, moles or cancer, blood vessels are widely distributed in the epidermis [20]. Transdermal delivery requires not only drug penetration through the stratum corneum, but also its diffusion through all these skin layers for proper efficiency.

### 2.2. Drug Diffusion Through the Skin

**Figure 2:**
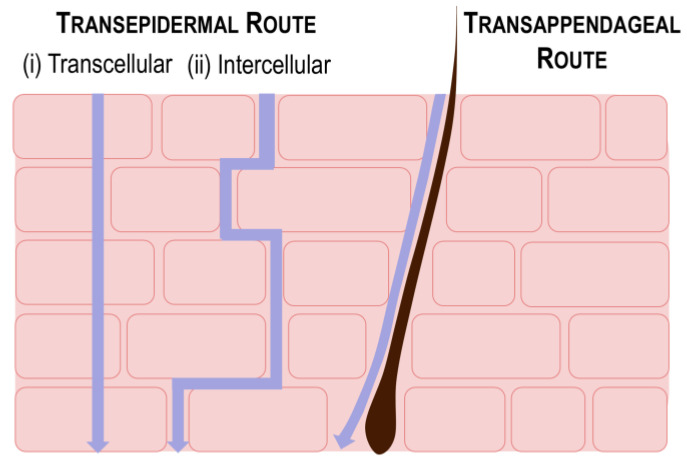
Scheme depicting the routes of drug diffusion through the skin. Each pathway is represented by a purple arrow below each label.

Effective drug penetration requires passage through the stratum corneum, the outermost layer of the epidermis. There are primarily two established pathways by which pharmacological agents can penetrate this barrier (Figure 2):

#### 2.2.1. Transepidermal Route

The transepidermal pathway involves the movement of substances across the cellular matrix of the skin [9,21]. This pathway can be divided into (i) transcellular and (ii) intercellular mechanisms. Transcellular permeation involves the direct absorption of drugs into and through individual skin cells, which is particularly efficient for hydrophobic compounds due to the lipid-rich environment of the cell membranes. Conversely, intercellular absorption occurs through the extracellular matrix by navigating the interstitial spaces between adjacent cells. The intercellular matrix is highly lipophilic; therefore, this mechanism is also particularly favorable for hydrophobic drugs and is recognized as the predominant mode of dermal drug absorption.

#### 2.2.2. Transappendageal Route

The transappendageal route refers to drug penetration through skin appendages such as hair follicles and sebaceous glands [9,21]. This route is advantageous for the delivery of polar or ionizable drugs, as well as macromolecules that may be hindered by the dense structure of the stratum corneum. The presence of these appendages provides alternative pathways that facilitate the passage of larger and more complex drug molecules, thus expanding the range of compounds that can achieve effective dermal absorption.

### 2.3. Types of Permeation Enhancers for Drug Delivery Through the Skin

Permeation enhancers are defined as substances that temporarily modify the skin’s barrier properties, thereby facilitating increased absorption of active compounds without causing lasting damage to the skin itself. By employing these enhancers, industries can markedly enhance the efficacy of their formulations, ensuring that active ingredients reach the intended depth in the skin or bloodstream in sufficient quantities to achieve the desired therapeutic effect. Table 1 provides an overview of the relevant permeation enhancer technologies, with the aim of providing the reader with a context for unfamiliar definitions. It should be noted that a comprehensive examination of these methods is beyond the scope of this review and has been well-covered by other publications.

**Table 1:** Summary of relevant technologies utilized as skin permeation enhancers.

Technology	Definition	Mechanism/Advantages	Ref.
Passive Strategies			
Permeation Enhancers	Molecules that increase the permeability of the stratum corneum	– Function by modifying: (i) Lipids (extracting or damaging lipids) (ii) Proteins (modifying the conformation of keratin or corneodesmosomes) (iii) Partitioning the drug into the stratum corneum – May also increase drug thermodynamic activity – Low cost, scalable and easy incorporation into formulations	[22]
Hydrogels	Highly flexible three-dimensional polymeric matrixes with ability to carry drugs	– High biocompatibility – Provide a constant moisture environment and resemblance to natural tissue – May be responsive to stimuli, allowing controlled release	[23,24]
Nanocarriers	Nano-sized particle systems designed to encapsulate and transport therapeutic agents	– Drug encapsulation allows protection from degradation and controlled release – May be of different origins (lipid-based, cell-based, inorganic, polymeric, among others) – May attach to targeting ligands for improved specificity and accommodate multiple drugs for combination therapy – Can be easily incorporated in other formulations or systems	[25,26,27]
Nanocrystals	Nanosized crystals (100–1000 nm) composed of a pharmaceutical drug	– High drug loading (up to 100%) in combination with a small amount of stabilizer – Faster dissolution rate and higher saturation solubility – Better consistency in oral absorption and bioavailability – Improved patient compliance by reducing the number of oral doses needed	[28,29]
Microemulsions (100–400 μm) and Nanoemulsions (1–100 nm)	Colloidal systems composed of oil and water	– Sub-categories: (i) Oil-in-water (O/W) (ii) Water-in-oil (W/O) – May enhance the solubility of both hydrophilic (W/O) and hydrophobic (O/W) drug molecules – Interfacial area, faster absorption and better drug solubility and bioavailability comparing to conventional emulsions – Microemulsions show higher stability, while nanoemulsions often require a cosurfactant for stabilization	[30]
Active Strategies			
Electrical Enhancers	Application of current to generate transient modifications in the stratum corneum	– Sub-categories: (i) Iontophoresis (low and continuous—10 V/cm or below) (ii) Electroporation (high and intermittent—from 100 V/cm) – Temporary enlargement or creation of pores – Enable the permeation of ionized or neutral drug molecules	[31,32,33]
Ultrasound	Utilization of low-frequency ultrasound to improve drug delivery	– Sub-categories: (i) Non-cavitational – High frequencies (1–3 MHz) – Disturbance of the stratum corneum through thermal effects – Enhancement of small lipophilic compounds (ii) Cavitational (sonophoresis) – Low frequencies of 20 kHz to 150 kHz – Generation of microbubbles in a coupling medium that collapse and create waves that create microchannels on the stratum corneum	[34,35,36]
Microneedles	Micrometer-sized arrays of needles arranged on a small patch to creates micropores in the stratum corneum	– Sub-categories: (i) Solid (ii) Hollow (iii) Coated (iv) Dissolving – Lower pain compared to hypodermic needles – Easy self-application with minimized risk of infection	[37,38,39]
Needleless Injections	Injection of a high-speed liquid medication jet to the skin	– Sub-categories: (i) Spring-loaded jet injector (ii) Gas-powered jet injector – Reduced risk of contamination – Easier self-administration – Reduced needle-stick injuries; however, jet cause bruising	[40]
Thermal Ablation	Utilization of high heat to disrupt or remove the stratum corneum	– Sub-categories: (i) Moderate temperature (below 100 °C for long period) (ii) High temperature (over 100 °C for short period) – Heat source can be either chemical, laser or radiofrequency – Heat induces cell ablation and transient creation of microchannels or pores (around 50–100 µm)	[41,42]

## 3. Considerations of Skin Structure for Drug Delivery and Strategies for Enhancement

The skin is a complex organ consisting of multiple layers, each characterized by distinct cell types and biological components. Although it covers the entire body, the structural characteristics of the skin can vary widely, a factor that must be carefully considered in the context of drug delivery. This section will review the specific aspects of skin structure and physiology relevant to drug delivery, highlighting the variations observed in different anatomical regions or during aging.

### 3.1. Hair Follicles

The surface of the skin is composed of pores, each containing a canal that gives origin to the hair follicles and sebaceous glands. Hair follicles are the most abundant in the scalp, forearms, legs, and genitalia. In axillary and genital regions, hair follicles may also contain apocrine sweat glands [43]. Hair follicles contain hairs, which are keratinized filaments composed of dead cells. The medulla represents the central core of the hair, which is circumscribed by the cortex. The cortex is a layer of compressed, keratinized cells that is covered by an outer layer of very hard, keratinized cells known as the cuticle. Morphologically, the hair follicle is constituted of three parts: the infundibulum, the isthmus, and the bulge (top to bottom) [44]. In the infundibulum, the outermost layer is impermeable. The permeability increases at the lower infundibulum, thereby facilitating drug penetration. In the isthmus region, the hair follicle is enclosed by an outer root sheath, which serves as a reservoir for stem cells for the regeneration of the skin and hair, and an inner root sheath, which plays a role in shaping the hair during its growth. At the bulge area, the dermal papilla contains hair bulge stem cells and an arrector pili muscle, which is responsible for erecting the hair upon stimulation. The bulb is densely vascularized, with a network of blood vessels that facilitate the delivery of nutrients to the growing hair. This proximity to blood vessels underscores the importance of pores as vital components of the skin, particularly in the context of transdermal drug delivery. In addition, since hair follicles extend to the dermis, or sometimes into the hypodermis, they facilitate better passage of certain drugs through the transappendal route. This route is especially relevant for transdermal delivery of nanocarriers such as lipid nanoparticles, liposomes, and polymeric nanoparticles [45]. These systems can be designed to release drugs over extended periods, effectively utilizing the follicles as long-term reservoirs [46]. The enhanced penetration of active compounds derived from nanocarriers can be attributed to the structure of the cuticular hair, which functions as a ratchet, propelling particles deeper into the hair follicle. In fact, research indicates that drugs can remain within the hair follicles for several days, providing a sustained therapeutic effect [47]. The unique anatomy of the hair follicle’s stratum corneum, which is thinner and more permeable than that of the surrounding skin, allows for transfollicular diffusion and direct transport into the viable skin layers.

The hair follicle is a notorious target for the delivery of drugs targeting hair regrowth. For example, nanocarriers loaded with the anti-hair loss drug minoxidil showed better accumulation in hair follicles compared to a commercially available minoxidil solution [48]. In addition, microneedles and chemical enhancers have also been utilized for this purpose [45]. Dissolving microneedles have been suggested to elevate Wnt signaling and promote hair follicle regrowth. Moreover, microneedles containing stem cell-derived nanocarriers and UK5099, a mitochondrial pyruvate carrier inhibitor, have been shown to stimulate hair growth and pigmentation. In addition, microneedles encapsulated with minoxidil also showed a positive prognosis in hair loss treatment. Furthermore, formulations containing minoxidil, a topically applied drug for hair loss, typically contain cosolvents as chemical enhancers that aid in the water solubility of this compound; therefore, improving its penetration. However, since these cosolvents may cause skin irritation, minoxidil hydrogels have been suggested as a possible alternative without these side effects and good therapeutic efficiency [49]. In addition, hair follicle regeneration is primarily driven by the activation of hair follicle stem cells [50]. Transdermal drug delivery strategies focusing on their stimulation offer a promising approach to enhance hair follicle regeneration in cases such as wounding. Microneedles integrated with conditioned media have shown great potential in promoting angiogenesis in hair follicle regions, thereby improving blood supply and regeneration capacity [51]. In addition, a platelet-rich plasma (PRP) gel has demonstrated the ability to stimulate hair follicle regeneration by activating dermal papilla cells, which play a key role in hair growth [52]. The transdermal delivery of pharmaceutical agents may prove beneficial in the treatment of conditions associated with inadequate hair follicle vascularization, such as androgenetic alopecia, a genetic disorder that causes the progressive loss of scalp hair. Finasteride, an SRD5A2 inhibitor for treating androgenetic alopecia, is associated with various side effects when administered orally. A recent study found that co-delivering finasteride with ferulic acid—a natural phenolic acid known for its vascular remodeling and anti-inflammatory properties—in active pharmaceutical ingredient ionic liquids based on choline can enhance the solubility of both compounds [53]. This approach improves their permeation and retention in hair follicles, promoting cellular uptake and facilitating hair regrowth. In fact, a recent study has demonstrated that hydrogel-forming microneedles loaded with PHA nanoparticles loaded with vascular endothelial growth factor (VEGF) and the hair loss drug Ritlecitinib promoted hair follicle angiogenesis and improved the immune microenvironment around the hair follicle [54]. In addition, alopecia areata is a condition that leads to loss of hair in some of the areas of the body. This is an autoimmune condition that may be triggered by environmental factors such as stress, or illness, or occur as a side effect of chemotherapy. A recent study highlighted the promise of phospholipid-calcium carbonate hybrid nanoparticles for the follicular delivery of Tofacitinib citrate, a JAK inhibitor that is effective in treating baldness but is associated with side effects when taken orally. These nanoparticles were found to inhibit apoptosis in mouse hair follicles and promote hair growth [55]. In addition, another study found that polymeric and lipidic nanoparticles effectively co-entrapped minoxidil and betamethasone for the topical treatment of alopecia areata, significantly enhancing follicular targeting. Notably, lipid nanoparticles provided a tenfold increase in minoxidil penetration and delivered higher concentrations of betamethasone in hair follicles compared to polymeric nanoparticles and control formulations, suggesting that these nanoparticles could improve treatment adherence while minimizing side effects associated with oral corticosteroids [56].

However, as a powerful drug reservoir, the hair follicle is also very useful for the delivery of other compounds. For example, curcumin, an herbal extract commonly used for wound healing, reduces inflammation and promotes cell proliferation by interacting with pro-inflammatory chemokines and cytokines, thereby enhancing epithelial regeneration, fibroblast proliferation, and collagen deposition. However, curcumin has low water solubility and bioavailability. A recent study demonstrated that curcumin-loaded micelles embedded in a microcurrent cloth resulted in increased accumulation in hair follicles, leading to higher wound closure rates, enhanced collagen regeneration, and elevated levels of cytokines such as IL-6 and IL-10, all while improving transdermal performance [57].

Moreover, hair follicles also contain sebaceous glands situated in the mid-dermis and attached to the follicle. These glands secrete sebum, which provides lubrication for the hair and skin. These structures are relevant for the transdermal delivery of drugs to target sebum-related disorders such as acne. For example, polymeric nanoparticles loaded with anti-acne drugs have been proposed to improve acne treatment by penetration through the hair follicle and accumulating within the pilosebaceous unit and hair follicles [58]. Furthermore, active mechanisms have been demonstrated to enhance drug permeation through the follicular route by reducing the viscosity of sebum. For example, a study demonstrated that the application of heat in combination with chemical penetration enhancers facilitated the delivery of the anti-acne medication isotretinoin via hair follicles [59]. In addition, a research group has recently developed niosomes loaded with anti-acne drugs tretinoin and bicalutamide to enhance follicular targeting for acne vulgaris treatment. The optimized formulation demonstrated significant accumulation in hair follicles, suggesting that the niosomal co-delivery system could be a promising topical treatment option for acne management while minimizing systemic side effects [60]. Moreover, tazarotene is a compound utilized for the treatment of inflammation-associated skin diseases such as acne vulgaris or plaque psoriasis. Tazarotene-loaded PLGA nanoparticles showed enhanced follicular and skin delivery from nanoparticles compared to solution [61]. Furthermore, azelaic acid micro/nanocrystals combined with ultrasound demonstrated improved penetration into hair follicles, exhibiting anti-inflammatory and antibacterial effects by inhibiting pro-inflammatory factors and targeting *Cutibacterium acnes* [62]. This highlights the potential for optimizing transdermal drug delivery by utilizing the distinctive characteristics of hair follicles. While they occupy a relatively limited area in comparison to the broader expanse of non-porous skin, hair follicles are of particular significance for targeting conditions affecting the follicles themselves or associated dermal appendages.

### 3.2. Facial Skin

**Figure 3:**
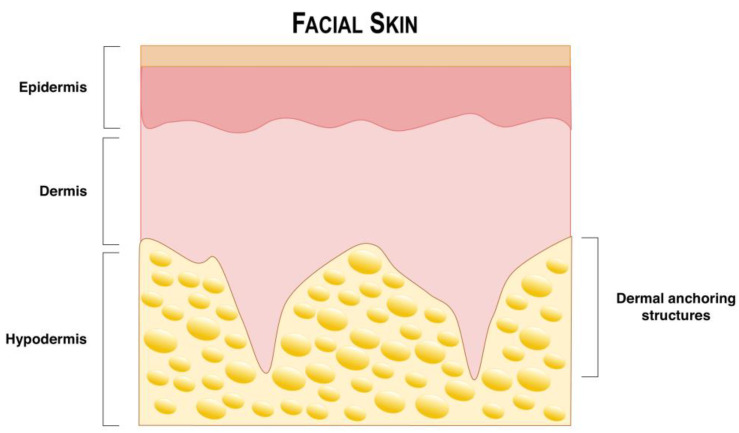
Dermal anchoring structures in facial skin (Adapted from Ezure et al., 2015 [65]).

In facial skin, the stratum corneum is thinner due to the presence of fewer layers of terminally differentiated keratinocytes and is generally associated with higher levels of transepidermal water loss (TEWL) and higher lipid contents, leading to a poorer barrier function [63]. Despite that, facial skin is considerably variable according to each area. A study analyzing facial skin thickness in various areas of the face found that the thickest facial skin is at the corners of the mouth, while the thinnest is at the lateral forehead [64]. In terms of the epidermis, the chin has the thickest layer, whereas the nasolabial fold has the thinnest. Regarding the dermis, the corners of the mouth have the thickest layer, and the lateral forehead has the thinnest. In light of the variable thicknesses of facial skin, it may be beneficial to consider parameters such as microneedle length or defining specifications for active delivery methods when developing drug delivery approaches to target this region. It is important to note that the study did not analyze the thickness of the eyelids. However, transdermal drug delivery to this area would present a greater challenge due to the frequent motion of eyelid skin. Moreover, a sonography study by the Sisheido Research Center identified the presence of “dermal anchoring structures” beneath the facial skin, connecting the dermal layer to the hypodermis (Figure 3) [65]. These protrusions exhibit a distinctive orientation of fibers within the dermal matrix, with a vertical alignment that contrasts with the horizontally oriented fibers that are typical of the skin. It is anticipated that the vertically oriented fibers may serve as anchoring elements that reinforce the overall structure of the skin, a process that is known to decline with age. These anchoring structures may affect transdermal drug delivery in the face; thus, the location and function of these protruding structures have the potential to be important for the development of delivery systems made through the face such as cosmetics. Moreover, some regions of the face may exhibit larger pores associated with the presence of bright tubular structures encircling hair follicles and enlarged hair shafts [66]. The face is also one of the locations of the body that accounts for higher sebum production. Genetic predisposition may lead some individuals to have hyperproduction of sebum, which may pose an advantage for the absorption of lipophilic drugs. In addition, sebum production may also be influenced by hormonal changes, as high levels of androgens and testosterone may stimulate sebum production or increase sebaceous gland sensitivity. Gender may also be implicated in this, as men usually display larger sebaceous glands compared to women. Therefore, when considering drug delivery methodologies to facial skin, it is important to consider parameters, such as the thickness of the region and the individual skin type of each person.

### 3.3. Glabrous Skin (Palmo-Plantar Regions)

**Figure 4:**
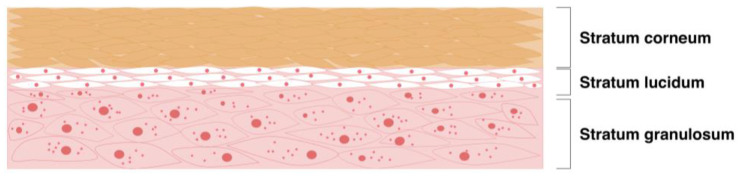
Scheme depicting the stratum lucidum typically present in the skin of hands and feet.

Glabrous skin, such as of the palms of the hands and plantar surface of the feet, is defined as smooth without the presence of hairs. This type of skin is notable for its thickness and resilience, enabling it to withstand considerable mechanical stress and provide vital support to the body while standing up [63]. Moreover, a microscopic analysis revealed that the stratum corneum of plantar skin is 16 times thicker than that of non-plantar skin, indicating a significant difference in the structural composition of these two types of skin [67]. While human epidermis typically varies between 60 and 100 μm of thickness, this thickness may be up to 600 μm in palmo-plantar regions. Moreover, distinctively from other skin areas, the epidermis of palmo-plantar regions possess an unique layer designated as the stratum lucidum, or transparent zone (Figure 4) [12]. The stratum lucidum is situated between the stratum granulosum and stratum corneum and exhibits a translucent appearance under microscopic examination. It is a thin layer typically composed of two to three layers of cells, with flattened keratinocytes representing the main component [12]. The interior is filled with eleidin modified with keratohyalin. The function and synthesis of this particular layer of skin remain unclear. It may be the result of the constant pressure exerted on the keratinocytes, which causes the cells to compact and induces the modification of the synthesized keratin proteins. These areas are also more prone to the development of pressure-related hyperkeratosis such as corns, calluses, warts, hand eczema or palmoplantar psoriasis. In addition, in the dermal layer, collagen fibers are thicker and show a distinct orientation from non-plantar skin, being oriented tangentially to the epidermis to aid in protection from mechanical stress [67]. In addition, the amount of lipids is much lower in these regions, making these areas drier and more prone to develop complications such as dermatitis in occupations requiring extensive mechanical stress. Moreover, despite the considerable thickness, the palmo-plantar regions demonstrate a notable degree of transdermal water loss, which is comparable to that observed in lesions such as acute dermatitis. Additionally, these regions exhibit a high abundance of sweat glands [63,68,69]. In that sense, the utilization of lipophilic drugs in these areas may be preferable. The aforementioned structural and physiological alterations pose significant challenges to transdermal drug delivery in these regions, particularly with regard to the thickness of the skin. Therefore, drug delivery in these regions may not be optimal, or if necessary, there is a need to develop specialized approaches.

### 3.4. Aged Skin

**Figure 5:**
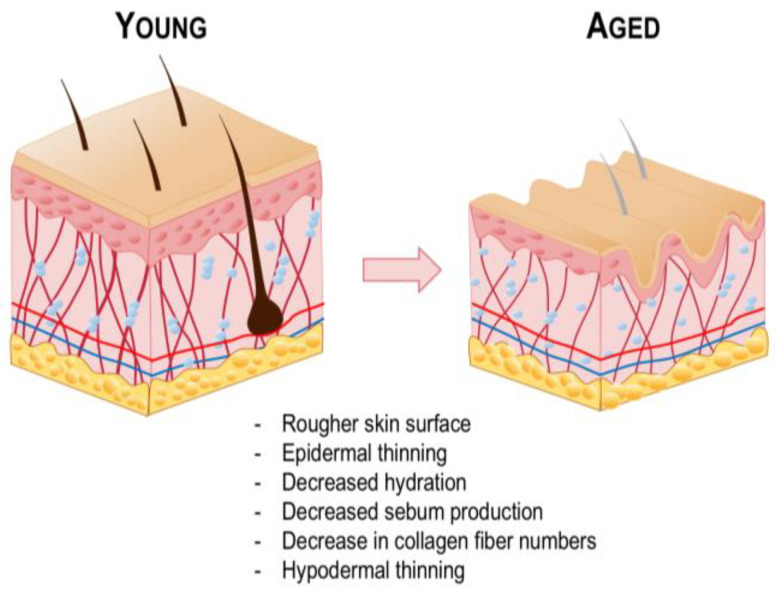
Graphical comparison of the various characteristics of young and aged skin.

As the aging process advances, a discernible reduction in the functional capacity of skin cells occurs, which may have impacts on the efficacy of transdermal drug delivery systems (Figure 5) [70]. This phenomenon can be attributed to intrinsic aging, which is a natural aspect of the human life cycle. Additionally, extrinsic factors, such as prolonged sun exposure or air pollution, can further intensify this process [71,72,73,74,75]. The cumulative effects of these factors may lead to structural and physiological changes in the skin, including decreased cellular turnover, reduced barrier function, and altered dermal composition. These changes can influence the permeability and absorption of transdermally administered drugs.

In the epidermis, the ability of basal keratinocytes to undergo division and differentiation is compromised, resulting in a reduction in the thickness of the stratum corneum and a decline in the normal process of desquamation [76]. As a result, aged skin presents higher roughness and scaliness [77]. Basal keratinocytes display variations in their size, shape and color, and the interface between the epidermis and dermis appears flattened [78]. Melanocytes become dysfunctional, and melanogenic activity decreases, resulting in a paler appearance [79,80]. However, melanocytes in sun-exposed areas demonstrate a tendency towards pigmentary changes, such as the formation of “aged spots”, including melasma or senile lentigo [81,82,83]. The hydration level of the stratum corneum is decreased, as amino acids generated in aged skin show higher hydrophobicity [84]. Despite that, a study in human patients found a low correlation between the subject’s age and stratum corneum hydration. Moreover, although sebaceous glands enlarge with age, the rate of sebum production declines due to reduced cellular turnover [85]. Consequently, surface lipid levels diminish with age. Given that the absorption process is susceptible to alterations in the concentration of skin lipids, a decline in lipid content may result in a reduction in the effective delivery of hydrophilic compounds. In such cases, lipophilic drugs may be preferable to enhance delivery efficiency. However, it is crucial to consider other factors, such as the intrinsic physiological differences between men and women, as the decline in androgen levels during menopause significantly impacts sebum production, potentially leading to more pronounced effects in women.

In the dermis, the rate of collagen degradation increases, and its biosynthesis decreases [86]. In young skin, collagen fibers are abundant, tightly packed, and well-organized. In contrast, aged skin exhibits fragmented and coarsely distributed collagen fibers, resulting in the thinning of this layer [87,88]. This structural deterioration leads to skin wrinkling and a loss of elasticity. Furthermore, prolonged sun exposure results in the accumulation of glycosaminoglycans (GAGs), which are associated with dermal hydration. However, their deposition is abnormal, occurring in the superficial dermis, which may be correlated to the drier skin status observed in aged individuals [89]. Moreover, hair follicles lose their function with aging, and hair becomes thinner and scarcer [90]. Furthermore, in contrast to other adipose tissue reservoirs, the hypodermis undergoes a reduction in thickness with age, which contributes to the overall skin thinning associated with dermal alterations [91,92]. It is important to note that disparate rates of aging across different body parts may occur, especially in areas that were subjected to chronic sun exposure, which can result in an uneven composition of the skin.

In elderly patients, transdermal drug delivery can be very advantageous. For example, the reduction in skin thickness that occurs with aging may be beneficial; however, it is important to consider the specific characteristics of each individual’s skin, including the potential for callus formation. Moreover, a considerable proportion of elderly patients may experience dysphagia or reduced venous access [84]. Furthermore, patient compliance may be enhanced since elderly individuals frequently take concomitant medications. Consequently, it is advantageous to employ methodologies that diminish the number of medications while prolonging the duration of drug action. Nevertheless, the complexity of self-administration associated with certain transdermal drug delivery modalities, such as patches, should be considered, as should the potential for patients to neglect their treatment regimen, a common occurrence with oral medications. Overall, the elderly have significant potential as a target population for transdermal drug delivery. However, it is essential to consider the unique characteristics of each patient and to develop personalized formulations that address their specific medical needs.

## 4. Considerations of Skin Pathology for Drug Delivery and Strategies for Enhancement

When developing effective drug delivery systems, skin pathology plays a critical role in determining how well therapeutic agents can penetrate and be absorbed through the skin. Various conditions significantly alter the skin’s structural integrity and barrier function. Understanding these pathological changes is essential for designing drug delivery systems that are effective in diseased skin. Additionally, utilizing strategies like permeation enhancers, microneedles, or advanced formulations can optimize drug delivery in pathological conditions by enhancing drug penetration, while ensuring higher bioavailability, patient compliance, and controlled release [93]. This section discusses considerations of various skin diseases and explores how these systems can support therapeutic interventions.

### 4.1. Atopic and Psoriatic Skin

**Figure 6:**
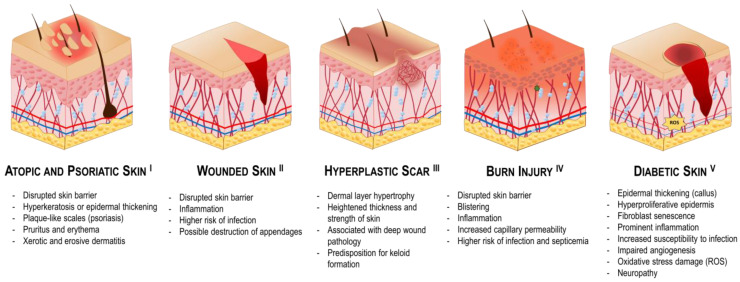
Graphical comparison of the characteristics of various skin conditions.

Atopic dermatitis and psoriasis affect a great portion of the population (Figure 6, I). Atopic dermatitis consists of a long-term inflammation of the skin associated with dryness and itchiness, with a global prevalence of 2.6%, as per a 2023 report, being more prevalent in females [94]. Psoriasis is an autoimmune disease characterized by the appearance of silver plaque-like lesions due to keratinocyte overgrowth, affecting 2–3% of the world population, according to a 2019 report [95]. Both conditions may present with analogous symptoms, including pruritus and erythema, as well as xerotic and erosive dermatitis with hyperkeratosis.

Though these conditions do not necessarily require transdermal drug delivery, various permeation enhancers associated with these systems may be beneficial to target these conditions. For instance, maintaining adequate dermal hydration through the use of hydrogels, emollients, or moisturizers can also help alleviate dryness and reduce the risk of exacerbations. In addition, since topical corticosteroids and calcineurin inhibitors may cause irritation with long-term use, the application of approaches such as nanocarriers may allow a safer and more effective delivery [96]. In fact, the development of advanced smart patches with controlled drug release is a prominent area of research for the treatment of atopic or psoriatic skin. Technologies such as responsive nanoparticle-embedded or microneedle-embedded hydrogel patches containing therapeutics are some examples of recent innovations [97,98,99,100,101]. As chronic inflammatory skin conditions, atopic and psoriatic skin often show heightened immune responses and metabolic disturbances [102,103]. In these cases, the usage of transdermal approaches may be particularly beneficial to locally target the bloodstream and regulate these mechanisms. The utilization of these systems enables the direct delivery of anti-inflammatory and immunomodulatory drugs, such as corticosteroids, calcineurin inhibitors, or biologics, through the skin. This approach allows for the modulation of immune activity with greater efficacy by maintaining constant blood concentrations and circumventing the fluctuations in drug levels that are characteristic of oral and injectable drug supplementation [104].

The barrier disruption associated with these conditions may be advantageous for drug absorption. However, it is important to note that in these conditions are often associated with a broken skin barrier, leading to potentially greater systemic exposure. While this can enhance the therapeutic effects of a given treatment, it also increases the risk of systemic side effects or toxicity, especially when potent drugs such as corticosteroids or analgesics are used. It is, therefore, essential to exercise caution when administering doses and monitoring the effects of such treatments in order to ensure that the benefits of localized treatment are not outweighed by the potential for unintended systemic absorption. In fact, a recent study found better drug retention in psoriatic skin due to the disrupted skin barrier associated with a higher transepidermal water loss (TEWL) values and lower electrical resistance [105]. The same report also found that enhancement techniques such as iontophoresis can aid in drug permeation, but the increased sensitivity of diseased skin can pose a significant risk of irritation, so precautions should be taken to avoid further irritation or damage, and the parameters of such enhancement techniques should be well controlled. It is also important to note that environmental factors such as temperature, humidity, or air pollution can increase the likelihood of irritation [100,101,106,107,108,109].

However, in some instances, atopic and psoriatic skin may present with epidermal hyperplasia associated with hyperkeratosis, which must be considered additionally in such cases. Hyperkeratosis refers to an increased thickness of the stratum corneum due to overproduction of keratin [110]. In addition to atopic dermatitis and psoriasis, this phenomenon may also manifest as a consequence of other conditions, including chronic physical pressure or friction to the skin, genetic mutations such as ichthyoses and keratoderma, squamous cell carcinoma, nutritional deficiencies, or even as a result of various pharmacological agents, including chemotherapeutics, among others. Careful consideration of these factors ensures more effective and targeted treatment strategies.

### 4.2. Wounded Skin

Wounding results from the disruption of the skin integrity due to the impact of external mechanical factors (Figure 6, II). This complex and dynamic process involves three overlapping but distinct phases: inflammation, tissue formation, and remodeling [111]. The inflammatory phase involves hemostasis and the recruitment of neutrophils and macrophages to initiate tissue formation. Interactions between endothelial cells and fibroblasts form a vascularized extracellular matrix (ECM) that provides structural support and promotes angiogenesis to supply nutrients and oxygen to the wound. As the wound heals, re-epithelialization and contraction occur simultaneously. The final phase, remodeling, begins with the apoptosis of macrophages and myofibroblasts. Over time, the type III collagen matrix is remodeled into the disordered type I collagen characteristic of scar tissue. Wound healing strategies should be non-toxic, enable effective delivery of therapies to the wound site, and protect these therapies from premature degradation [111]. In addition, the type and stage of the wound (acute vs. chronic, superficial vs. deep) must be considered, as these will impact both drug choice and delivery system design.

For instance, chronic wounds are associated with persistent inflammation, which can compromise blood circulation at the wound site, increase white blood cell count and reactive oxygen species, and show higher predisposition for infection, worsening other symptoms and hindering healing [112,113]. In these cases, transdermal drug delivery systems can target the affected area while also modulating systemic responses. The delivery of anti-inflammatory or pain-relieving medications without the usage of oral administration may be beneficial in these cases. Additionally, transdermal systems reduce the need for injections or frequent dressing changes, minimizing infection risks by delivering drugs while providing a protective barrier against bacterial contamination. For example, the integration of materials such as nitrile rubber and natural extracts, including *Chromolaena odorata*, into transdermal patches has been investigated as a means of facilitating wound healing. The nitrile rubber backing serves as an effective vehicle for the delivery of therapeutic agents while maintaining an optimal microenvironment for healing [114]. In addition, microneedles combined with smart responsive materials have been surging as a painless hybrid wound dressing combining hypodermic needles and transdermal patches that can tackle the complexity of the wound microenvironment by improving inflammation, angiogenesis, oxygen levels, pH, and temperature [115]. Another study found that short-term high-intensity ultrasound significantly enhances the proliferation, migration, and extracellular matrix production of human dermal fibroblasts in wound healing models. This mechanical stimulation activates key signaling pathways, suggesting that ultrasound could be an effective strategy to improve skin regeneration and wound healing outcomes [116]. In atopic or psoriatic skin, the disrupted skin barrier associated with open wounds may improve delivery efficiency, but the skin may become more sensitive and prone to irritation, and the risk of infection is increased. In such situations, it is important for wound dressing to incorporate antimicrobials and constant moisture, such as that provided by hydrogels or advanced patch systems [23,117,118,119].

Furthermore, in superficial wounds, the regenerative process typically progresses efficiently as the underlying cells undergo mitosis, differentiate, become keratinized, and are gradually displaced outward by the ascending layers of the epidermis. However, deep wounds pose significant challenges to regeneration due to severed collagen, elastin fibers, and potential damage to skin appendages like hair follicles and sweat glands. Moreover, deep wounding may often lead to blood vessel damage, hindering healing and requiring the targeted administration of antibacterial agents to prevent systemic infection.

An examination of the regenerated skin from deeper wounds reveals that while the thickness of the epidermis remains consistent due to its ongoing desquamation, the dermal layer becomes hypertrophied with densely packed fibers. Consequently, these wounds frequently result in scar formation, which can become hypertrophic and occasionally develop into keloids (Figure 6, III) [120]. Due to the heightened thickness and strength of the skin surface, drug delivery in areas like this is challenging and may benefit from the use of enhancers. To target hypertrophic scars, microneedles have been proposed as a solution to address these challenges [121,122,123,124]. For example, dissolving microneedles has been utilized to deliver triamcinolone acetonide, a corticosteroid commonly used in the treatment of keloids [125]. A clinical trial demonstrated that these microneedles effectively dissolve within the skin, allowing for localized drug delivery with minimal pain and the potential for self-administration by patients. Moreover, a dissolvable microneedle system containing quercetin-loaded biomimetic nanoparticles was developed for the targeted treatment of hypertrophic scars, demonstrating enhanced efficacy through the regulation of Wnt/β-catenin and JAK2/STAT3 pathways [126]. The system also exhibited superior mechanical strength and stability, making it a promising candidate for skin disease treatments. Additionally, a study demonstrated that the transdermal administration of betamethasone, when combined with fractional laser treatments, resulted in a significant improvement in the management of hypertrophic scars [127]. This combination therapy not only enhances drug delivery but also promotes new collagen formation, which is crucial for scar remodeling. Similarly, the use of 5-fluorouracil delivered via ethosomal gels has demonstrated efficacy in reducing scar formation in animal models, indicating the potential of these strategies for more efficient drug delivery [128].

### 4.3. Burn Injury

Burns occur when the skin is injured by sources such as heat, electricity, radiation, lasers, or chemicals (Figure 6, IV). Although the healing process progresses in a similar manner as conventional wounds, burn wounds may differ substantially [129]. For instance, the surface area affected is often more extensive in cases of burns, and there are systemic differences, such as increased capillary permeability and extravasation of fluid due to heat, as well as an elevated risk of infection and septicemia. Depending on the severity, burns may be superficial (first-degree), partial (second-degree), full-thickness (third-degree) or very deep (fourth-degree) [130]. Superficial burns affect only the epidermis, while partial burns reach the low epidermis or high dermis. The necessity for surgical intervention is dependent upon the extent of the damage. Full-thickness burns affect the dermal layer, and very deep burns damage as deep as the muscle tissues or bones.

Permeation enhancers have emerged as a significant approach for managing burn wounds due to their ability to provide controlled and sustained release of therapeutic agents [131]. The necessity of these approaches in burn care is primarily driven by the acute pain associated with these injuries, which often requires effective analgesics for relief. Moreover, since burns can impact multiple body surfaces and are often associated with a modified dermal structure and enhanced vascularization in burned regions, transdermal drug delivery can be beneficial for systemic administration [132]. For instance, the delivery of analgesics, such as opioids, continuously ensures consistent plasma levels and improved pain management without the need for frequent dosing. Additionally, burn wounds are highly susceptible to infections, which can impede healing and lead to severe complications. By facilitating the systemic delivery of antibiotics, adequate therapeutic concentrations are maintained in the bloodstream, thus enhancing the effectiveness of infection control measures.

Burn wound dressing is commonly required as a temporary cover to protect the wounds from further injury and external infections. A wound dressing should be non-adhesive, antimicrobial, easy to take off and put on, flexible, non-bulky, and highly absorbent [133]. Hydrogels have gained significant attention as a good wound dressing technology given their high water content, biocompatibility, non-toxicity, and ease of application. Moreover, microneedles allow for minimally invasive drug administration, enabling larger molecules to penetrate the skin without significant discomfort. In that sense, hydrogel microneedle patches are capable of incorporating therapeutic agents within nanocarriers, thereby enhancing the efficacy of drug delivery by facilitating deeper penetration into the wound site while retaining wound dressing abilities. The controlled release of therapeutics from hydrogel microneedle patches loaded with bioactive agents has been demonstrated to facilitate improved healing outcomes in deeper tissues affected by burns [132]. Drugs such as antimicrobial agents, antioxidants, anti-inflammatory agents, analgesics, and growth factors, which are relevant for burn healing, can be successfully incorporated into these systems, rendering them a versatile approach for this condition. Furthermore, the utilization of natural products, such as a nanoethosomal gel derived from Ashitaba leaves, has been demonstrated to expedite the healing process of burn wounds in animal models, thereby substantiating the efficacy of permeation systems in topical burn therapy [134]. In addition, the use of snakehead fish powder in nanoemulgel formulations has been characterized for its stability and potential as a permeation enhancer [135]. The non-invasive nature of these systems is particularly beneficial in burn care, where minimizing additional trauma to the skin is crucial.

### 4.4. Diabetic Skin

Diabetes is a chronic metabolic disorder that is characterized by elevated blood glucose levels (Figure 6, V) [136]. These elevated levels result from insufficient insulin production, insulin resistance, or a combination of both. Prolonged hyperglycemia can originate a multitude of complications throughout the body, including notable alterations in the skin, which are commonly referred to as diabetic skin [137,138]. Dermatological conditions such as dryness, infections, and impaired wound healing are hallmarks of diabetic skin. In particular, diabetic skin frequently displays xerosis, or marked dryness, due to diminished sebum production and impaired skin barrier function. Furthermore, patients may be prone to developing skin infections more frequently due to the immunosuppressive effects of diabetes. Diabetic skin is also linked to neuropathy, which arises from hyperglycemia and metabolic imbalance caused by oxidative stress [139]. Other noteworthy alterations include a reduction in collagen synthesis, impaired circulation, and a shift in inflammatory responses, all of which can markedly impact the skin’s overall health and integrity. Diabetic skin primarily manifests on the plantar surface of the foot, including the ball, the tips of the toes, and the lateral of the foot, collectively known as the diabetic foot [140]. Additionally, diabetic skin may manifest on other areas of the body, including the scalp, ankles, trunk, or genitalia. Ulcers are frequently circular in shape and characterized by a puncture-like appearance with a surrounding callus.

To enhance diabetic skin wound healing, recent advancements related to permeation enhancers have been proposed to improve healing efficiency or the delivery of therapeutic agents. For example, various hydrogels have been recently developed for diabetic wound healing. For instance, a self-healing, injectable polypeptide-based hydrogel was developed for the controlled release of bioactive exosomes, showing promising results in promoting diabetic wound healing and achieving complete skin regeneration [141]. Additionally, a glucose and MMP-9 dual-response temperature-sensitive hydrogel was developed, exhibiting self-adaptive properties for deep diabetic wounds, with controlled release of insulin and celecoxib, leading to reduced inflammation, enhanced wound protection, and accelerated healing [142]. Also, a dual-drug-loaded, polysaccharide-based self-healing hydrogel has been developed to address the challenges of diabetic chronic wound healing by incorporating metformin for rapid release and curcumin-loaded nanoparticles for sustained release [143]. This multifunctional hydrogel promotes antibacterial activity, enhances tissue adhesion, and accelerates wound healing through re-epithelialization, collagen deposition, angiogenesis, and wound contraction, offering a promising solution for regenerative medicine. Importantly, diabetes frequently impairs angiogenesis, leading to inadequate blood flow to the wound site. Recently, several microneedle approaches have been developed to stimulate angiogenesis in diabetic wounds, including the stimulation of angiogenesis. For example, microneedles loaded with Prussian blue nanozymes and vascular endothelial growth factors have been developed for diabetic wound healing, showing positive results in promoting angiogenesis, antioxidant activity, and antibacterial effects [144]. Additionally, double-layer microneedles incorporating tetracycline hydrochloride in the tip and recombinant human epidermal growth factor (rh-EGF) in the substrate have demonstrated strong antibacterial properties by rapidly releasing tetracycline to fight infections, while rh-EGF promotes angiogenesis, cell migration, and tissue regeneration, effectively reducing inflammation and accelerating wound healing [145]. Similarly, microneedles loaded with deferoxamine have been shown to enhance angiogenesis by stimulating vascular growth while also providing antibacterial effects and promoting collagen deposition, contributing to faster and more efficient diabetic wound repair [146]. Another innovative microneedle dressing, based on glucose-responsive insulin-releasing hydrogel, has shown effective wound healing, improved blood glucose control, and decreased inflammatory reactions [147]. Moreover, a recent study developed a microneedle bandage functionalized with dopamine-coated hybrid nanoparticles to regulate reactive species generation in diabetic wound environments, showing effective biofilm eradication, anti-inflammatory effects, and accelerated wound healing [148]. In addition, a magnesium organic framework-based microneedle patch has been developed to enhance diabetic wound healing through transdermal delivery and combination therapy [149]. This multifunctional patch releases magnesium ions to promote cell migration and endothelial tubulogenesis, while gallic acid acts as a reactive oxygen species scavenger to provide antioxidative benefits alongside antibacterial effects from the graphene oxide-silver nanocomposites, resulting in significant improvement in wound healing in a diabetic mouse model. Exosomes derived from pioglitazone-pretreated mesenchymal stem cells have been developed, showing enhanced angiogenesis, improved migration and tube formation of endothelial cells, and accelerated wound healing. These exosomes promoted collagen deposition and ECM remodeling, effectively activating the PI3K/AKT/eNOS pathway, making them a promising cell-free therapeutic strategy for diabetic wound treatment [150]. These studies emphasize the necessity to develop effective systems that cater specifically to the needs of diabetic wounds, which require specific considerations to improve therapeutic outcomes.

### 4.5. Melasma

**Figure 7:**
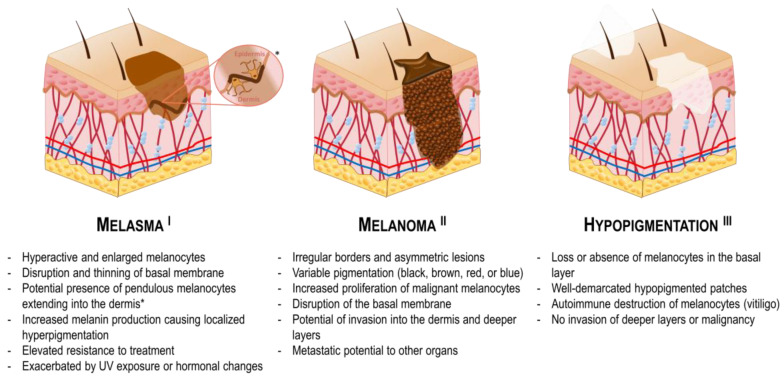
Graphical comparison of the characteristics of various pigmentary skin conditions. The asterisk indicates a representative illustration of pendulous melanocytes.

Melasma is a prevalent dermatological condition characterized by the appearance of brown or grayish hyperpigmented lesions on the skin (Figure 7, I) [151,152]. This condition is caused by an increase in melanin production and is more prevalent in sun-exposed areas, such as the face. Melasma is typically multifactorial, with a number of potential contributing factors. These include genetic predisposition, hormonal changes, certain medications, environmental triggers or UV radiation. In melasma, the melanocytes are characterized by hyperactivity and often present with an enlarged morphology. This overactivity results in the formation of visible dark patches on the skin surface, which are particularly evident due to the uneven distribution of melanin in the stratum corneum, the outermost layer of the epidermis. In more severe cases, melanocytes may also be pendulous, protruding into the dermal layer, where melanin is engulfed by dermal melanophages. This deeper pigment deposition often results in a bluish or grayish hue, which renders the condition more resistant to treatment. Most melasma lesions show damage in the basal membrane, with a substantial number of single, non-aggregated melanosomes [152]. Furthermore, there is evidence suggesting a role for increased vascularization in the areas affected by melasma, which might contribute to the condition, although this aspect remains poorly understood.

Permeation enhancers have emerged as a promising approach to improving the treatment of melasma. These systems facilitate the penetration of therapeutic agents through the skin barrier, thereby enhancing their bioavailability and efficacy. For example, topical agents such as hydroquinone, tretinoin, corticosteroids, and other drugs are frequently employed in the treatment of melasma [153,154]. However, these agents often encounter limitations due to inadequate dermal penetration and potential adverse effects [155,156]. For instance, triple combination therapy with these agents has been demonstrated to induce cutaneous irritation and erythema in some patients [155]. In addition, dryness, burning sensation and pruritus may also occur [157]. The utilization of enhancement systems allows for the regulation of active ingredient concentration, which may result in a reduction in adverse effects while improving efficacy. Recent developments in nanocarrier and microneedle systems are being investigated with the aim of improving the delivery of therapeutic agents to treat melasma, which may prove particularly beneficial when this condition involves both the epidermis and dermis [158,159,160]. For example, a transethosomal patch was developed for transdermal delivery of tranexamic acid to treat melasma, showing superior performance compared to conventional creams [161]. In addition, a paeoniflorin-glycyrrhizic acid complex transethosome gel was developed to enhance transdermal delivery and anti-melasma efficacy of paeoniflorin and glycyrrhizic acid [162]. The gel showed sustained release, high permeability, and significant effects in preventing melasma by reducing skin inflammation and collagen loss. It also decreased oxidative damage and down-regulated melanin-related proteins, making it a promising treatment for melasma. Moreover, dissolving microneedles containing tranexamic acid showed enhanced delivery, higher bioavailability and better melanin reduction [163]. The combination of tranexamic acid with licorice extract further improved efficacy, and the microneedles demonstrated good stability under stress conditions. Another study developed curcumin-loaded permeation enhancer nanovesicles, which showed improved skin permeation and reduced hyperpigmentation in a rabbit model [31]. Also, aspasomes containing Mg ascorbyl phosphate, a Vitamin C derivative, showed high drug permeation and skin retention [164]. When tested in patients, a formulated aspasomal cream containing was evaluated clinically as an effective treatment for melasma, demonstrating its potential as an effective and side-effect-free treatment.

The multifactorial nature of melasma necessitates innovative strategies for effective treatment. Advances in drug delivery systems and permeation enhancers show promise in improving therapeutic outcomes, offering hope for more effective management of this skin disorder.

### 4.6. Melanoma

Melanoma is a malignant form of skin cancer that arises from melanocytes (Figure 7, II) [165]. The management of melanoma frequently entails surgical excision; however, for advanced or metastatic cases, systemic therapies, such as immunotherapy and targeted therapy, are indispensable. In clinical settings, melanoma typically manifests as an evolving, asymmetrical, hyperpigmented patch with irregular borders. The coloration of melanoma can vary considerably, with hues ranging from black and brown to red or blue, which can complicate its diagnosis [166]. In addition to its visual characteristics, melanoma is known for its aggressive nature and propensity for metastasis, often spreading to lymph nodes and other organs if not detected early [167]. Factors such as sun exposure, skin type, and genetic predisposition play a significant role in the development of melanoma, emphasizing the importance of regular skin examinations for early detection [168].

Local drug delivery is a crucial component of melanoma treatment, particularly for addressing localized lesions and complementing other therapeutic approaches [169]. Melanoma can exhibit resistance to conventional treatments, emphasizing the need for the exploration of innovative therapeutic strategies. Moreover, early-stage melanoma typically has a favorable prognosis, while advanced stages present considerable challenges, necessitating a multifaceted approach to management that integrates both local and systemic therapies. Since the skin’s natural barrier limits the penetration of many therapeutic agents, especially larger or poorly soluble molecules, effective delivery methods become necessary for drugs to reach the deeper layers of the skin or the tumor cells. In fact, recent studies have unveiled innovative techniques aimed at significantly enhancing the topical delivery of melanoma therapies. For example, a study presented a novel paintable oligopeptide hydrogel loaded with paclitaxel-encapsulated cell-penetrating peptide-modified transfersomes designed to enhance transdermal drug delivery for the treatment of cutaneous melanoma [153]. By embedding these transfersomes in a hydrogel, paclitaxel demonstrated prolonged skin retention and improved drug penetration through the stratum corneum, efficiently delivering paclitaxel to the tumor site. This innovative system showed promising results in slowing tumor growth in a melanoma mouse model, offering an effective alternative to conventional systemic chemotherapy. Moreover, a recent study reported on a nanoparticle-based photosensitizer that enhances the effectiveness of photodynamic therapy in melanoma treatment [170]. This technology was shown to increase ROS production, leading to melanoma cell death and the release of signals that trigger immune responses. Dendritic cells exposed to these treated cells showed markers of immune activation, indicating the potential of this approach to both destroy cancer cells and boost the immune system’s response against tumors. In addition, a niosomal nanoplatform was developed to enhance the solubility, skin permeation, and anticancer effects of ozonated olive oil for melanoma treatment [171]. By encapsulating ozonated olive oil in niosomes, skin absorption and sustained release were improved compared to free oil. When tested on melanoma cells, this formulation exhibited double the anticancer efficacy, highlighting its potential as a natural therapeutic strategy for melanoma. Additionally, another study employed a chrysin-loaded nanoemulgel for the treatment of melanoma, showing improved drug solubility, skin absorption, and anticancer effects [172]. The gel showed strong mechanical properties and better absorption through the skin, leading to enhanced melanoma cell killing. This approach showed a more effective and safer way to deliver chrysin, reducing both dosage and treatment frequency. Furthermore, two formulations of nanostructured lipid carriers, comprising either oleic acid or pomegranate oil, were developed to facilitate the topical delivery of ibrutinib for the treatment of early-stage melanoma [173]. These formulations demonstrated effective control of drug release and enhanced skin penetration, with those containing oleic acid exhibiting superior performance compared to those with pomegranate seed oil.

These novel strategies demonstrate the promising advantages and continued interest of topical delivery in melanoma management. However, the heterogeneous characteristics of melanoma lesions, coupled with natural variations in skin thickness and integrity, present significant challenges in formulating standardized treatment strategies. Therefore, future research may benefit from the development of tailored formulations and delivery strategies that address these complexities, ultimately aiming to improve therapeutic outcomes for patients with melanoma.

### 4.7. Hypopigmentation

Skin hypopigmentation refers to a condition in which the skin develops lighter-colored patches due to decreased melanin production (Figure 7, III) [174,175]. Hypopigmentation patches may originate from disorders such as vitiligo, albinism, pityriasis alba, tinea versicolor, nevus depigmentosus, or conditions such as post-inflammatory hypopigmentation or hypopigmented mycosis fungoides, among others [176,177]. The most common form of hypopigmentation is vitiligo, a chronic skin condition characterized by the loss of pigment-producing melanocytes, resulting in distinct depigmented patches on the skin. Vitiligo can affect individuals of all skin types and is believed to arise from a combination of genetic, autoimmune, and environmental factors that contribute to the autoimmune destruction of melanocytes, where the body’s immune system attacks these cells [178]. In addition, the incidence of vitiligo is higher in people with diabetes. Most hypopigmented macules involve the loss or absence of melanocytes in the basal layer, leading to well-demarcated areas that appear lighter than the surrounding tissue. Notably, hypopigmentation is typically superficial, with no invasion of deeper skin layers or associated malignancy, distinguishing it from conditions related to hyperpigmentation. The depigmented areas of the skin are often more susceptible to sunburn and may exhibit varying sensitivity compared to the surrounding pigmented skin. Moreover, hypopigmentation can significantly impact psychological well-being, as the visible changes may lead to social stigma and emotional distress. Therefore, effective management of hypopigmentation is essential for improving the quality of life for affected individuals.

Various studies have been focusing on the development of novel delivery enhancement strategies to ameliorate hypopigmentation. For example, a recent study evaluated the dermato-pharmacokinetics of transethosomes and hybrid ethosomes/nanostructured lipid carriers, demonstrating that the hybrid formulation was effective for both dermal and transdermal delivery of tofacitinib citrate, leading to significant repigmentation in a vitiligo mouse model while reducing systemic IFN-γ levels and minimizing potential side effects [179]. Additionally, the study developed a novel hyaluronate-integrated gel-core oleosomes nanovesicular system that significantly enhanced the dermal availability of berberine, demonstrating improved antioxidant and anti-inflammatory effects in a vitiligo mouse model while ensuring effective localized treatment with minimal systemic side effects [180]. Moreover, a study investigated the effects of nonablative fractional lasers on the transdermal uptake of topicals used to treat dermatologic conditions, such as vitiligo, alopecia, and cancerous lesions [181]. The findings revealed that laser pretreatment significantly enhanced the retention of the drug bimatoprost and improved the permeation of 5-fluorouracil and minoxidil, highlighting the potential of laser-assisted therapies to optimize treatment efficacy while emphasizing the need to balance effective absorption with minimal thermal side effects. Another study evaluated the potential of transethosomes and hybrid ethosomes/nanostructured lipid carriers to deliver Tofacitinib citrate, a Janus kinase 1/3 inhibitor drug, revealing that these strategies promoted repigmentation in a vitiligo mouse model [179]. In addition, a study applied fractional ablative CO_2_ laser treatment to vitiligo patients with acral lesions, which are often resistant to standard therapies [182]. The laser treatment was followed by a five-day course of 5-fluorouracil cream and led to significant improvements in repigmentation. Although not life-threatening, skin hypopigmentation can significantly affect individuals’ quality of life due to its complex underlying causes and psychological repercussions, making it a vital area of ongoing research. Recent advancements in permeation enhancement methods show promise in enhancing repigmentation while minimizing side effects. Continued research in these areas is crucial for developing effective treatments that restore skin pigmentation and improve overall well-being.

## 5. Considerations Regarding Skin Models for Dermatological Research

**Figure 8:**
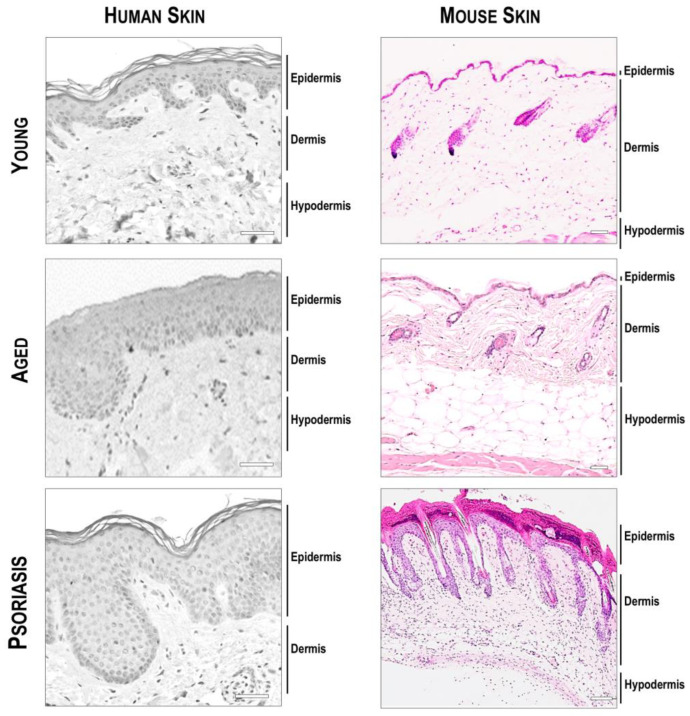
Histological comparison of young, aged, and psoriatic human and mouse skin. Scale bar = 50 μm. Human forearm skin: Young, 24 years old; Aged: 74 years old; Psoriasis, 34 years old. Mouse dorsal skin: Young, 10 weeks old; Aged, 80 weeks old; Psoriatic model, 10 weeks old. Patient biopsies were obtained after approval by the Institutional Review Board of the Dongguk University Ilsan Hospital and were conducted according to the Declaration of Helsinki principles. Mouse experiments were approved by the Institutional Animal Care and Use Committee of Semyung University (IACUC; Approval No. smecae 20-04-01) and were performed according to the animal testing guidelines. Mouse psoriatic model was induced with 10% hydrogen peroxide treatment for 24 h.

Proper evaluation of the efficiency of topical and transdermal drug delivery systems is crucial to understanding their penetration and pharmacological abilities. Due to the various restrictions associated with the usage of in vivo human skin, the usage of skin models has become standardized (Table 2). A wide range of skin models are currently available for this purpose, including in vivo animal and chimeric models, ex vivo human and animal models, artificial models and reconstructed models [183]. First, the usage of in vivo animal models has been very widespread in research, including animals such as zebrafish, rodents, rabbits, and pigs. Among these, mice are the most commonly utilized due to the ease with which they can be obtained and manipulated. In the context of drug delivery, they permit the induction of a plethora of disease models or genetic mutations that can be used to mimic human skin conditions and facilitate the evaluation of several parameters concomitantly, for which the methodologies are already well standardized. While there are certain similarities between mouse skin and human skin, the number of differences is considerable, rendering a direct comparison difficult. With regard to the epidermis, analogous alterations can be observed with aging and in psoriatic skin (Figure 8). The aged epidermis is not well differentiated in both cases and often exhibits hyperkeratized areas in comparison to the young epidermis. Additionally, in psoriatic skin, it is also possible to observe that there is hyperkeratized or undifferentiated epidermis with inflammation. However, as an animal with fur, the epidermal layer of mice is notably thinner, being composed of only a few layers, while human skin possesses many layers. Furthermore, the stratum corneum is less robust in mice, which facilitates cutaneous absorption. In addition, wound healing progresses distinctly in mice and humans, as mice skin depends on contraction for healing, while humans rely more on re-epithelization [184]. These differences between mice and humans should be considered while testing transdermal delivery products.

In comparison, rat skin offers superior advantages due to its closer resemblance to human skin in terms of thickness [185,186]. Rat skin can provide a more realistic model for human skin in dermatological and transdermal studies, allowing for more accurate extrapolation of results to human applications. However, mice might be a preferable option for many researchers due to their lower cost and easier maintenance. Porcine skin is highly comparable to human skin due to its similar properties, including thickness, hair density, and epidermal turnover time, among other factors [187]. However, working with porcine skin in vivo is labor and cost-intensive, posing significant challenges in terms of logistics and management. Additionally, these models may give rise to ethical concerns, a growing issue in society. Zebrafish have been categorized as a promising model for psoriasis due to their genetic similarity to humans, fast embryonic development, and advanced genetic tools like CRISPR for precise gene manipulation [188]. They also have a fully functional innate immune system and share key structural features with human skin, allowing the creation of relevant psoriasis models that offer real-time insights into disease mechanisms and therapeutic targets. However, this is only appropriate for evaluating the molecular mechanisms of psoriasis, as fish possess an epidermis that is very distinct from humans and has different barrier properties. Zebrafish offer unique advantages for early screening but are less relevant in direct comparison to human skin.

**Table 2:** Summary of the main distinctions and considerations between human and animal skins commonly used in research.

	**Human**	**Mouse**	**Rat**	**Porcine**	**Zebrafish**
Stratum corneum	Thick (10–20 μm)	Thin (~5 μm)	Thick (~25 μm)	Thick (20–26 μm)	Absent
Epidermis	Thick (60–100 μm)	Thin (~30 μm)	Thin (~60 μm)	Thick (30–140 μm)	Thin
Dermis	Thick (1–4 mm)	Thin (~400 μm)	Thick (~3 mm)	Thick (~2 mm)	Thin (<200 μm)
Hair follicles	Sparse	Dense	Dense	Sparse	Absent
*Panniculus carnosus*	Vestigial	Present	Present	Present	Absent
Permeability	Moderate	High	High	Moderate	High
Healing mechanism	Re-epithelization	Contraction	Contraction	Re-epithelization	Re-epithelization
Main advantages	-	– Genetically well-characterized – Low cost and easy to handle – Short lifecycle	– Similar skin structure to humans in terms of epidermis and dermis – Larger size makes sampling easier compared to mice	– Closest to human skin – Good model for drug absorption and diffusion studies	– Transparent skin allows for easy in vivo imaging and monitoring of drug effects – Low maintenance costs and fast reproduction rate – Suitable for early-stage, high-throughput screening
Main disadvantages	-	– Thin skin and high permeability – Significant structural differences in the stratum corneum and hair follicle density – Less accurate for predicting human drug absorption	– Higher permeability than human skin – Larger surface area requirements and higher maintenance costs than mice – Limited availability of advanced genetic models compared to mice	– Larger size requires more space and higher maintenance costs – Ethical concerns with larger mammals – Slower breeding and maturation rates compared to rodents	– Poor correlation with human skin
References	[189,190]	[190,191]	[185,186]	[187]	[188,192,193]

Regarding ex vivo skin models and in vivo chimeric models (such as human skin xenografts on mice), their low availability, high variability, and technical difficulty make them unattractive for researchers. Moreover, a great portion of the existing synthetic models are based on simple artificial membranes that serve for studies of drug diffusion and permeation. In addition, in silico models can also be utilized to predict drug permeation and diffusion using mathematical algorithms [194]. While cost-effective and reproducible, these methods lack the complexity of real skin, and the array of evaluation parameters is limited. The aforementioned arguments show the necessity to develop methods that are more cost-effective, practical, and ethical while better reproducing the features and composition of human skin. Recent advances in bioengineering have focused on the development of 3D bioprinting and organ-on-a-chip skin models. With the progress in inkjet printing technology, 3D bioprinting skin models have gained prominence due to their low cost, customizability, scalability, high efficiency, reproducibility, and ability to generate complex structures [195,196,197]. In these models, multiple layers can be stacked with bioink made from cells of the various skin layers, which may be embedded in a wide array of substrates. In addition, given their customizable nature, they also show potential for the modeling of skin diseases. Skin-on-a-chip models, on the other hand, integrate skin cells cultured in microfluidic devices that mimic the natural physiological environment by continuously supplying nutrients, oxygen, and other essential components [198,199,200]. These models have proven useful for a wide range of drug delivery assessments, including testing for toxicity, efficacy, wound healing, inflammation, aging, and stress. These innovative approaches show great promise; further optimization and development are necessary, as these models are still relatively new and require more research to be able to mimic the complexity of real skin more specifically. Nonetheless, the current landscape suggests that there is considerable potential for continued exploration in this field, and many exciting new technologies are anticipated in the near future.

## 6. Conclusions

The skin varies significantly across different body regions, displaying distinct features that influence how drugs are absorbed and distributed. Designing effective delivery systems requires careful consideration of the skin’s unique properties, such as its thickness, permeability, and regional differences, as well as the individual’s overall health. While permeation enhancers have been used for long, the field continues to evolve rapidly, driven by technological advancements. Integrated smart technologies, which allow for more personalized and adaptive drug delivery, exemplify this modernization and highlight the ongoing interest from researchers. Additionally, novel skin models for testing transdermal systems are being developed to provide more ethical, economical, and reproducible alternatives to traditional methods. These models are crucial for accurately evaluating the efficacy and mechanisms of emerging technologies and must continue to be refined to support the research community’s needs.

## Data Availability

Data sharing is not applicable.
